# Improved fully convolutional neuron networks on small retinal vessel segmentation using local phase as attention

**DOI:** 10.3389/fmed.2023.1038534

**Published:** 2023-03-01

**Authors:** Xihe Kuang, Xiayu Xu, Leyuan Fang, Ehsan Kozegar, Huachao Chen, Yue Sun, Fan Huang, Tao Tan

**Affiliations:** ^1^The University of Hong Kong, Pokfulam, Hong Kong SAR, China; ^2^The Key Laboratory of Biomedical Information Engineering of Ministry of Education, School of Life Science and Technology, Xi’an Jiaotong University, Xi'an, Shaanxi, China; ^3^College of Electrical and Information Engineering, Hunan University, Changsha, Hunan, China; ^4^Faculty of Technology and Engineering (East of Guilan), University of Guilan, Rudsar-Vajargah, Guilan, Iran; ^5^Faculty of Applied Sciences, Macao Polytechnic University, Macau, Macao SAR, China; ^6^Electrical Engineering, Eindhoven University of Technology, Eindhoven, Netherlands

**Keywords:** segmentation, unsupervised enhancement, retinal vessel, local phase, orientation scores

## Abstract

Retinal images have been proven significant in diagnosing multiple diseases such as diabetes, glaucoma, and hypertension. Retinal vessel segmentation is crucial for the quantitative analysis of retinal images. However, current methods mainly concentrate on the segmentation performance of overall retinal vessel structures. The small vessels do not receive enough attention due to their small percentage in the full retinal images. Small retinal vessels are much more sensitive to the blood circulation system and have great significance in the early diagnosis and warning of various diseases. This paper combined two unsupervised methods, local phase congruency (LPC) and orientation scores (OS), with a deep learning network based on the U-Net as attention. And we proposed the U-Net using local phase congruency and orientation scores (UN-LPCOS), which showed a remarkable ability to identify and segment small retinal vessels. A new metric called sensitivity on a small ship (*Se_sv_*) was also proposed to evaluate the methods’ performance on the small vessel segmentation. Our strategy was validated on both the DRIVE dataset and the data from Maastricht Study and achieved outstanding segmentation performance on both the overall vessel structure and small vessels.

## 1. Introduction

In recent years, many research works have revealed that retinal fundus images can provide much helpful information, which is related to multiple diseases, such as Age-related Macular Degeneration (AMD), Glaucoma, Diabetic Retinopathy (DR) arteriosclerosis, and hypertension (1). Therefore, retinal image analysis is increasingly essential for computer-aided diagnosis (2).

Retinal vessels, as a part of the blood circulation system, have been proven to contain many essential biomarkers (1). The small retinal vessels (defined as vessels with a width less than 65 μM in this paper), including small-artery and small-vein, are much more sensitive to the blood circulation system lesion and have great significance in the early diagnosis and warning of diseases (1). Thus, the accurate segmentation of retinal vessel structures, tiny vessels, is a crucial part of the quantitative analysis of retinal images. Considering the complexity of retinal vascular trees, automatic segmentation is necessary to eliminate the cumbersome and time-consuming manual label processing. However, due to the low contrast between retinal vessels and background, the variation of vessel width, the complex geometric structure of small boats, and the severe background noise problem (1), that is a challenging task.

Most retinal vessel segmentation methods were focused on the performance of the overall retinal vessel structures and evaluated *via* global performance metrics like accuracy (Acc), sensitivity (Se), specificity (Sp), and area under the ROC curve (AUC). At the same time, the segmentation performance on the small vessels needed to receive more attention due to their small percentage in retinal images. A segmentation method with high sensitivity on small plates was required to identify and segment the small vessels from retinal images.

The recent works about retinal vessel segmentation can be classified into two categories, unsupervised and supervised methods.

Unsupervised retinal vessel segmentation methods, usually based on prior knowledge about the vessel structures in retinal images, include kernel-based methods, vessel tracking methods, model-based methods, etc. Kernel-based methods depend on the different specially designed filter kernels to detect the vessel structures. Azzopardi et al. (3) proposed an approach based on the combination of shifted filter response (COSFIRE) to see the bar-shaped facilities and achieved rotation invariability. Zhang et al. (4, 5) designed the left-invariant derivative (LID) filter on orientation scores (LID-OS) and achieved superior performance on the segmentation of crossings and bifurcations. Vessel tracking methods detect vessel structures by tracing the ridges of retinal vessels. Bekkers et al. (6) described two vessel tracking approaches, edge tracking in orientation scores (ETOS) and multi-scale vessel center line tracking in orientation scores (CTOS). De et al. (7) proposed a two-step tracing approach and gave a top way to address the problem of tracing with crossover. Model-based methods apply deformable models to identify vessel structures. Al-Diri et al. (8) and Zhao et al. (9) designed an infinite active contour model for the vessel segmentation task. With much simpler processes, unsupervised methods are usually faster than supervised ones. Besides, manually labeled ground truths are unnecessary for unsupervised methods. Thus the most troublesome step can be avoided. However, due to the complexity of vessel structures, one unsupervised way is often only able to cope with some possible vessel structures. Therefore unsupervised methods usually have poorer performance.

Supervised methods usually have better segmentation performance. Generally, a supervised learning algorithm produces a model which contains specific knowledge derived from the input images and manually labeled ground truths for the segmentation of retinal vessels. Fraz et al. (10) proposed the ensemble classifier of boosted and bagged decision trees and achieved *Acc*, *Se*, *Sp,* and *AUC* for 0.7406, 0.9807, 0.9480, and 0.9747 on the DRIVE dataset. Convolutional Neural Network (CNN) has dominated many computer vision tasks. With the ability to extract hierarchical features and take advantage of contextual information, CNN performs remarkably in medical image segmentation tasks. Ronneberger et al. (11) proposed the U-Net, a CNN specialized for biomedical image segmentation tasks. The U-Net conducts the convolution operator to extract features from original images and gets segmented photos directly *via* the up-sample operator. Wang et al. (12) used the U-Net in the retinal segmentation task and achieved an *AUC* of 0.9790 on the DRIVE dataset. The best vessel segmentation results were achieved in supervised ways (13). However, because of the reliance on manually labeled ground truths, laborious work is inevitable for supervised methods. Besides, for most deep learning approaches, massive labeled retinal images are required in network training, which makes things even trickier.

Notably, most of the proposed works mentioned above neglected the tiny vessels in retinal images, and none involved specific reports about the segmentation performance on small retinal vessels. No segmentation method or performance evaluation method for small plates was proposed.

This paper proposed a novel method called the U-Net using local phase congruency and orientation scores (UN-LPCOS). We combined the unsupervised methods of local phase congruency (LPC) and orientation scores (OS) with the deep learning network modified from the U-Net. The LPC, proposed by Kovesi et al. (14), showed a superior ability to enhance small vessels in retinal images. The LPC was invariable to the image contrast through analyzing images in the frequency domain and had high sensitivity to the small plates. The OS method was also adopted. With outstanding performance on complex vessel structures, such as crossings and bifurcations, the OS method can improve the robustness of our method. The retinal vessels were enhanced by LPC and OS, respectively. Then the original retinal images and two vessel-enhanced images were combined and served as the input of a U-Net-based deep learning network. The network produced the vessel probability score of each pixel. After thresholding, the binary images of vessel segmentation were obtained.

The proposed UN-LPCOS was validated on the DRIVE dataset (15) and the data from Maastricht Study (16). Some commonly used metrics, such as sensitivity (*Se*), specificity (*Sp*), accuracy (*Acc)*, and area under the ROC curve (*AUC*), were calculated and compared with other proposed methods.

A new evaluation metric, called sensitivity on a small vessel (Sesv), was defined to describe different methods’ abilities on small vessel segmentation. We also discussed the effect of different unsupervised vessel enhancement results in our practice and revealed the significance of LPC and OS in small vessel segmentation.

The main contributions in this paper were summarized as follows:

- We combined two unsupervised vessel enhancement methods, LPC and OS, with the modified U-Net. We proposed a novel vessel segmentation method called UN-LPCOS, which achieved outstanding performance on small retinal vessel segmentation.
- We were the first to evaluate the abilities of different methods on small vessel segmentation. We proposed a new evaluation metric, called sensitivity on a small vessel (*Se_sv)_*, to describe the performance of small retinal vessel segmentation quantitatively.

The rest of this paper was organized as follows. In section 2, some crucial methodologies involved in our method were introduced. The details of the experiments to validate our approach are displayed in section 3. And the experiment results are shown in section 4. Finally, further discussion was involved in section 5.

## 2. Methodology

**Figure 1:**
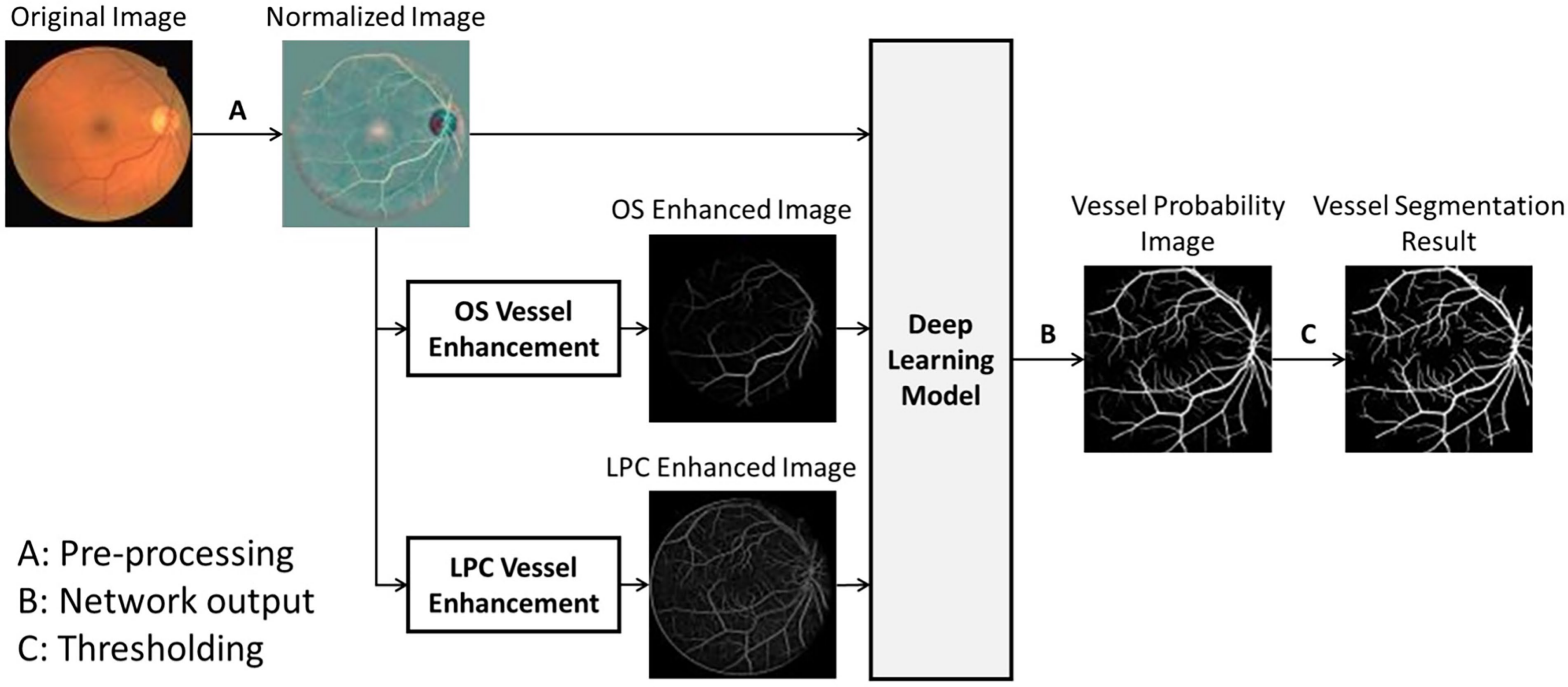
The overall process of UN-LPCOS, including unsupervised vessel enhancement and vessel segmentation.

The overall process of the proposed UN-LPCOS was presented in Figure 1, which contained three significant parts, image preprocessing, vessel enhancement, and vessel segmentation. The local luminosity normalization method was adopted in the image preprocessing to deal with retinal images’ local luminosity and contrast variation problem. Then the unsupervised vessel enhancement was conducted to highlight the vessels, especially the tiny and complex vessel structures in retinal images. In this paper, the LPC and OS were applied for vessel enhancement, and they significantly improved the ability of our method on small vessel detection and identification. The vessel-enhanced images combined with the preprocessed retinal photos served as the input of the deep learning network based on the U-Net. The network produced the vessel probability score for each pixel. After the thresholding, the binary vessel segmentation results were derived.

This section introduces some essential principles and methods adopted in the UN-LPCOS. The local luminosity normalization for the image preprocessing was presented first. Then we introduced two unsupervised vessel enhancement methods, local phase congruency and left-invariant derivative filter on orientation scores. Finally, the architecture of the deep learning network was illustrated.

### 2.1. Local luminosity normalization

The local luminosity and contrast variation problem are the significant interferences in retinal images due to the irregular retinal surface and non-uniform illumination. To overcome this issue, the local luminosity normalization method proposed by Foracchia et al. (17) was adopted for image preprocessing. It can be denoted below,

(1) $$N\left(x,y\right)=\frac{f\left(x,y\right)}{\frac{1}{n^{2}}\sum_{i=1}^{n^{2}}f\left(x_{i},y_{i}\right)}$$

where the N(x,y) represents the normalized pixel intensity at the position (x,y) , and the numerator is the original pixel value. The denominator is the arithmetic mean of the n×n neighbors pixels’ intensity around (x,y) , respectively (17).

### 2.2. Local phase congruency

Local phase congruency, proposed by Kovesi et al. (14), was a frequency domain image processing method with high sensitivity on small vessel structures. Unlike gradient-based methods, the LPC defined features based on the similarity of the local phase angles of different frequency components in images. Therefore, a dimensionless measurement of parts, invariable to the image contrast, was produced.

In this paper, the LPC of images was calculated through wavelets transformation. The original idea was denoted as *I*, while $M_{n,\theta }^{o}$ and $M_{n,\theta }^{e}$ represented the even and odd wavelet filters with the scale *n* and orientation θ , respectively. The image was convolved with the wavelet filters, and the filter responses were marked as $F_{n,\theta }$ and $H_{n,\theta }$ , which were written as (14),

(2) $$F_{n,\theta }=I*M_{n,\theta }^{e};$$

(3) $$H_{n,\theta }=I*M_{n,\theta }^{o}.$$

Based on that, the local spectrum amplitude $A_{n,\theta },$ and local phase angle $\phi_{n,\theta }$ of the image with corresponding scale and orientation were calculated as (14),

(4) $$A_{n,\theta }=\sqrt{F_{n,\theta }{}^{2}+H_{n,\theta }{}^{2}};$$

(5) $$\sin\phi_{n,\theta }=\frac{H_{n,\theta }}{A_{n,\theta }};$$

(6) $$\cos\phi_{n,\theta }=\frac{F_{n,\theta }}{A_{n,\theta }}.$$

And the weighted mean of the local phase angle with the orientation θ was defined as (14):

(7) $$\sin\bar{\phi_{\theta }}=\frac{\sum_{n}H_{n,\theta }}{\sqrt{{\left(\sum_{n}F_{n,\theta }\right)}^{2}+{\left(\sum_{n}H_{n,\theta }\right)}^{2}}},$$

(8) $$\cos\bar{\phi_{\theta }}=\frac{\sum_{n}F_{n,\theta }}{\sqrt{{\left(\sum_{n}F_{n,\theta }\right)}^{2}+{\left(\sum_{n}H_{n,\theta }\right)}^{2}}}.$$

Then the modified local energy of the image, which achieved more localization accuracy, was derived as:

(9) $$E_{\theta }=\underset{n}{\sum }A_{n,\theta }\left[\cos\left(\phi_{n,\theta }-\bar{\phi_{\theta }}\right)-\left|\sin\left(\phi_{n,\theta }-\bar{\phi_{\theta }}\right)\right|\right],$$

and the local energy was calculated in each orientation (14).

Noise interference was one of the most intractable problems in the LPC. Thus, the noise threshold was introduced to suppress the noise in the LPC results. Since the noise spectrum was flat, the smallest wavelet filter, with the most considerable bandwidth, obtained the most energy from the noise, and the noise threshold was derived from it. The magnitude of the most negligible wavelet response followed a Rayleigh distribution. Its median was the expectation (denoted as $\mu_{\min}$ ) of the noise distribution. The noise responses of filters with other scales and the response of the smallest filter were proportional to the bandwidth. Therefore, the noise threshold $T_{\theta }$ was given as follows:

(10) $$T_{\theta }=\underset{n}{\sum }\mu_{n,\theta }+k\sigma_{n,\theta },$$

where $\mu_{n,\theta },$ and $\sigma_{n,\theta }$ represented the estimation of the expectation and variance of the noise response of the filter with the scale of n and the orientation of θ , respectively (14).

Due to the smoothing operation, different frequency components in the image had extra significance. We applied the frequency weighting function to describe this difference. It was written as:

(11) $$W_{\theta }=\frac{1}{1+\exp\left(g\left(c-\frac{1}{N}\left(\frac{\sum_{n}A_{n,\theta }}{\varepsilon +A_{\max}}\right)\right)\right)},$$

where *c* was the cut-off value of the filter response spread and *g* was a gain factor that controlled the cut-off’s sharpness (14).

Finally, we obtained the expression of the LPC. It was notable that, since the features would present in any direction of images, we calculated and combined the results of different orientations. Thus, the LPC was denoted as,

(12) $$LPC=\frac{\sum_{\theta }\left[W_{\theta }{\left(E_{\theta }-T_{\theta }\right)}^{+}\right]}{\varepsilon +\sum_{\theta }\sum_{n}A_{n,\theta }}$$

where the small constant 𝜀 was provided to cope with the situation where the $\underset{\theta }{\sum }\underset{n}{\sum }A_{n,\theta }$ was tiny, and the ()^+^ meant that if the local energy were smaller than the noise threshold, the difference would be 0 (14).

The LPC orientation was also derived for the following vessel segmentation process to distinguish vessels from noise in the LPC results. LPC orientation was defined as the orientation with the maximum LPC value at each pixel, which was written as,

(13) $$\theta_{LPC}=\underset{\theta }{\mathrm{argmax}}\left(\frac{W_{\theta }{\left(E_{\theta }-T_{\theta }\right)}^{+}}{\varepsilon +\sum_{n}A_{n,\theta }}\right).$$

**Figure 2:**
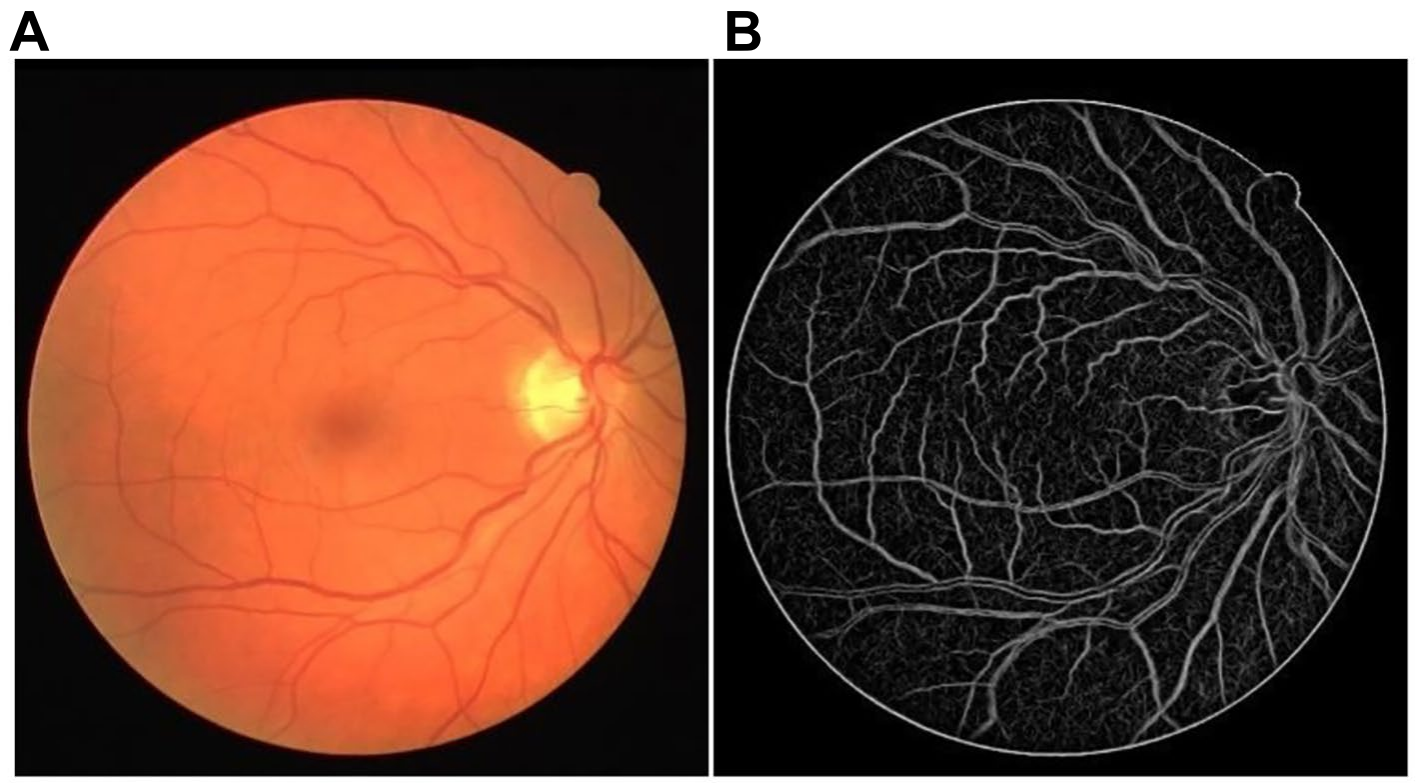
The example of LPC vessel enhancement result. **(A)** The original retinal image from the DRIVE dataset. **(B)** The LPC vessel enhanced the result.

Figure 2 shows one LPC vessel enhancement result of the DRIVE dataset. The small retinal vessels with low contrast were highlighted well and had similar feature intensity to the main retinal vessels. A low noise threshold was adopted to present as many small structures as possible, which leads to more noise in the LPC result. Fortunately, it would not influence the final vessel segmentation.

### 2.3. Left-invariant derivative filter on orientation scores

Due to the extremely complex structure, many segmentation methods lost their stability on retinal vessels. Many vessel segmentation approaches, based on the detection of tubular structures, failed on the bifurcation points and crossovers of retinal vessels. To overcome this problem and improve the robustness of the proposed UN-LPCOS, orientation scores (OS) was adopted to support vessel segmentation in this paper. More specifically, the left-invariant derivative filter on orientation scores (LID-OS), proposed by Zhang et al. (4), was involved.

The basic idea of the OS and LID-OS filters is described as follows. The original 2D image was disentangled into several orientation channels *via* anisotropic wavelets transformation, which was given as:

(14) $$U_{f}\left(\mathrm{x,}\theta \right)=\left(\bar{R_{\theta }\left(\psi \right)}*f\right)\left(x\right)=\int \bar{\psi \left(R_{\theta }^{-1}\left(y-x\right)\right)}f\left(y\right)dy.$$

Where $R_{\theta }\left(\psi \right)$ represented a set of anisotropic filters, and *f* represented the retinal image. The orientation scores of the image were denoted as $U_{f}\left(x,y\right)$ , which was derived through the convolving of, and *f* (4).

Then a set of unique filters called left-invariant derivative (LID) filters were applied to the orientation scores to enhance the tubular vessel structures. The LID-OS filters were constructed on the LID frame to ensure the Euclidean invariance, which was defined as,

(15) $$\left\{\partial_{\xi },\partial_{\eta },\partial_{\theta }\right\}=\;\left\{\cos\theta .\partial_{x}+\sin\theta .\partial_{y},\cos\theta .\partial_{y}-\sin\theta .\partial_{x},\partial_{\theta }\right\}$$

Based on the LID frame, the multi-scale rotating LID-OS filters were derived from the second-order Gaussian derivatives on orientation scores, and they were defined as:

(16) $$\Phi_{\eta ,norm}^{\sigma_{s},\sigma_{o}}\left(U_{f}\right)=\mu^{-2}\Phi_{\eta }^{\sigma_{s},\sigma_{o}}\left(U_{f}\right)=-\mu^{-2}\partial_{\eta }^{2}G_{\sigma_{s},\sigma_{o}}*U_{f},$$

where μ was a normalization factor, which was given by $\mu =\sigma_{o}/\sigma_{s}$ . The physical unit 1/ length kept the convolution results dimensionless and truly scale-invariant (4).

Finally, the 2D vessel enhanced image was reconstructed by taking maximum filter response in the orientation scores, and the final reconstruction output was written as:

(17) $$\gamma \left(f\right)\left(x\right)=\underset{\theta_{i}\in \frac{\pi }{N_{o}}\left\{1,\ldots ,N_{o}\right\}}{\max}\left\{\underset{\sigma_{s}\in S}{\sum }\Phi_{\eta ,norm}^{\sigma_{s},\sigma_{o}}\left(U_{f}\right)\left(x,\theta_{i}\right)\right\}$$

**Figure 3:**
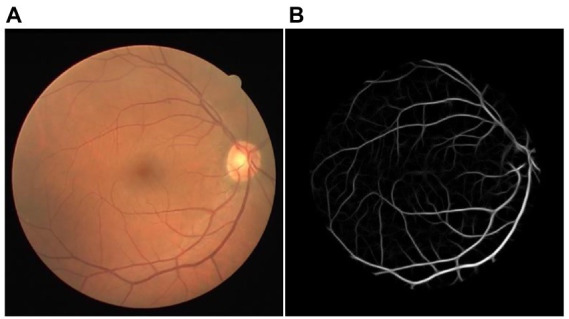
The example of LID-OS vessel enhancement result. **(A)** The original retinal image from the DRIVE dataset. **(B)** The LID-OS vessel enhanced the result.

One LID-OS vessel enhancement result of the DRIVE dataset was displayed in Figure 3. The bifurcations were enhanced with high quality. The enhancement result achieved outstanding vessel connectivity, and the noise was suppressed well (4).

### 2.4. Modified u-net

**Figure 4:**
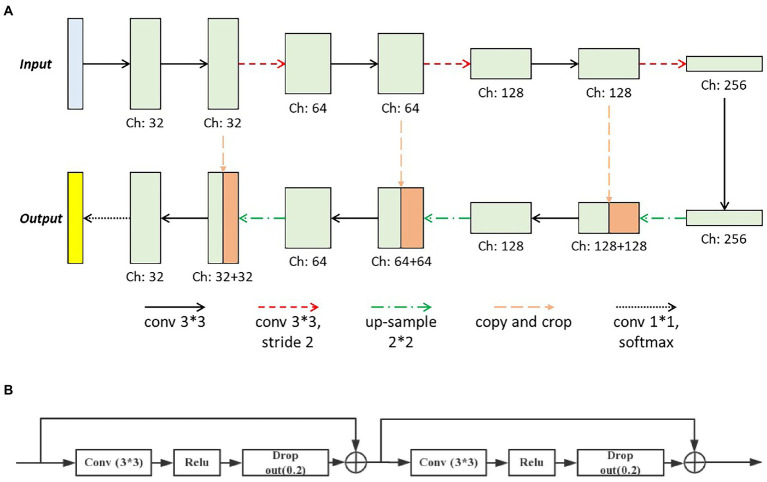
The basic architecture of **(A)** our deep learning network modified from the U-Net and **(B)** the modified convolution operation set.

The deep learning method was adopted in this paper to produce the final pixel-wise vessel probability image. And the preprocessed retinal photos combined with the vessel-enhanced results served as the input of the deep learning network. The basic architecture of our network was modified from the U-Net (shown in Figure 4A), consisting of two major parts, the encoder, and the decoder. The encoder extracted features from the input image through the hierarchical convolution operation. At the same time, the decoder did the de-convolution and up-sample procedure and produced the vessel probability image *via* the final softmax active function. The vessel probability image had the same size as the manual labeled ground truth and the input image. In each expansive operation of the decoder, the corresponding feature images from the encoder were concatenated with the feature images to be up-sampled. The deep learning model could be trained end-to-end with very little training data. It was crucial since only a few labeled retinal images were available (11).

Two significant modifications were introduced in our deep learning network. First, we modified the convolution operation sets in the original U-Net, which contained two repeated 3×3 convolutional layers and each followed by a rectified linear unit (ReLU). We introduced the identity short-cut, which was inspired by the ResNet, and the detailed structure of modified convolution operation sets was presented in Figure 4B. Besides, to reduce the loss of the local image feature of the small vessel structure, we replaced all max-pooling layers in the original U-Net with convolutional layers with two strides for the down-sampling. These modifications could accelerate the training process of the deep learning model and enable the model to better identify the small vessel’s features.

## 3. Validation and experiment

### 3.1. Dataset

In this paper, the proposed method was validated on three datasets, the DRIVE dataset, the data from the Maastricht Study and the UoA-DR (18) database. The DRIVE dataset consists of 40 retinal images, divided into two parts, a training dataset and a test dataset, and each piece has 20 photos. Every picture has a resolution of 565*584 pixels. The vessel structures of each image are annotated by two human investigators separately. Our deep learning model was trained on the training dataset and evaluated on the test datasets.

The data from Maastricht Study contains 600 retinal images, which are taken through the NIDEK AF230 with the resolution of 3,744 * 3744 and resized to 1024*1024 with small structures preserved. The population of the Maastricht Study contains 1,363 healthy subjects (NGM), 366 prediabetes subjects (preDM), and 610 type two diabetes subjects (T2DM). Experienced ophthalmologists from the ophthalmology department in the Maastricht Medical Center, Maastricht, Netherlands label the ground truth of the blood vessels for each image. And the dataset is divided into two parts, a training dataset with 400 photos and a test dataset with 200 illustrations. The Eindhoven University of Technology IRB exempted the study from IRB approval.

The UoA-DR database consists of 200 high-quality images captured using a Zeiss VISUCAM 500 fundus camera with a FOV of 45 and a resolution of 2,124 × 2056 pixels in JPEG format. The optic nerve head center, optic nerve head, macula, fovea, and the retinal vessels of all the 200 images in this database were manually segmented by a specialist ophthalmologist who acted as the first observer and by an optometrist as the second observer. The manually segmented features, such as retinal vessels, OD and fovea, for the 200 retinal images may be used to benchmark the performance of new ARIA methods in the future. This database can be downloaded for free with certain access rights mentioned in Ref. Like the MESSIDOR database, not all the images marked as high quality are of MSRI quality.

### 3.2. Evaluation measurements

Based on the manually labeled ground truths, the pixels of final vessel segmentation results were divided into four categories: The true positive (*TP*) represented the pixels that were labeled as vessels in ground truths and correctly identified in model outputs. The actual negative (*TN*) referred to the pixels labeled as non-vessels in ground truths and denoted as non-vessels in output images. The human investigator considered the false positive (FP) as the pixels labeled as non-vessels but classified as vessels in segmentation results. And the false negative (*FN*) represented the pixels that were denoted as vessels in ground truths but not identified in segmented images.

For comparison, we adopted some commonly used evaluation metrics, Sensitivity (*Se*), Specificity (*Sp*), and Accuracy (*Acc*), to evaluate the global performance of the proposed method on vessel segmentation. These metrics were given by:

(18) $$Se=\frac{TP}{TP+FN};$$

(19) $$Sp=\frac{TN}{TN+FP};$$

(20) $$Acc=\frac{TP+TN}{TP+TN+FP+FN}.$$

Besides, the receiving operator characteristics (ROC) curve and the area under the ROC curve (AUC) were also recorded to evaluate our method’s performance on vessel enhancement.

The evaluation metric, called sensitivity on small vessels (Sesv), was defined to describe different methods’ abilities in segmenting small retinal vessels. The pixels of small vessels were separated from the ground truth first, and they made up the ground truth of small vessels, which determined the range of pixels we cared about in the small vessel segmentation. In this paper, vessels with a width under 65 μM were defined as small vessels, which can be separated from the segmentation result and ground truth, respectively, using morphological opening operation. The kernel used for morphological opening can be round with a diameter of 65 μM. Two new pixel categories were proposed: true positive on small vessels (TPsv) and false negative on small vessels (FNsv). They represented the pixels labeled as vessels in the ground truths of small vessels and identified as vessels or non-vessels, respectively. The 𝑆𝑒_𝑠𝑣_ was defined as:

(21) $$Se_{sv}=\frac{TP_{sv}}{TP_{sv}+FN_{sv}}$$

The ROC curve on a small vessel (ROC_sv_) was derived from the *Se_sv_* versus the 1 − *Sp* concerning the varying threshold value *T_h_*. The area under the ROC_sv_ (*AUC_sv_*) was also calculated to evaluate the model’s performance on the minor vessel enhancement.

### 3.3. Experimental configuration

#### 3.3.1. Local phase congruency configuration

The LPC analysis in retinal images was conducted *via* 2D wavelet transform. With six different scales, a set of anisotropic Gabor wavelets were adopted to calculate the embodiment’s local amplitude and phase. The minimum wavelet length was 3, and each following wavelet length was multiplied by 2. There were 12 wavelets with different orientations for every size to detect the features in any image direction. The coefficient of standard deviations *k* for the noise threshold calculation was set as 3. And in the frequency weighting function, the cut-off value *c* and the gain factor *g* were set as 0.4 and 10, respectively. Besides, in the LPC calculation, the feature orientation at every pixel was also recorded, which was the direction where the maximum LPC value was obtained.

#### 3.3.2. Left-invariant derivative filter on orientation scores configuration

To apply the LID-OS in the vessel enhancement, retinal images were transformed into orientation scores. In this paper, the construction of orientation scores was implemented by convolving images with a set of rotated filters, precisely, the cake wavelets proposed by Duits et al. (19) They could be regarded as a set of quadrature filters, and the fundamental part represented the locally symmetric structures like ridges/lines. In contrast, the imaginary part responded to the antisymmetric structures like edges. Eight filter directions were uniformly selected. Then the second-order Gaussian derivatives in the LID frame were applied directly to the orientation scores of retinal images, and blood vessel structures were highlighted in each orientation layer. Therefore, the complex vessel structures, such as crossings and bifurcations, were enhanced well. The final vessel-enhanced results were reconstructed by obtaining the maximum filter response on all orientation layers.

#### 3.3.3. Training of deep learning network

Data augmentation was conducted first to generate enough training data. In this paper, the method of cutting was adopted. Small patches, of dimension 64*64, on both the DRIVE dataset and the Maastricht Study data, were sampled randomly from the original training images and corresponding ground truths. To give our network the ability to discriminate the edge of the field of view (FOV) from vessel structures, the patches partially or entirely outside the FOV were also selected. For each training epoch, 9,000 patches were obtained by randomly extracting 450 patches in each of the 20 DRIVE training images. The first 90% of patches (8,100 patches) were used for training, while the last 10% (900 patches) were used for validation. And for the data from Maastricht Study, 100 patches were extracted randomly from each training image, and we got a total of 40,000 patches from all 400 images. Similarly, the first 90% of patches were training data, and the other 10% were validation data. For both datasets, all patches were reselected in each training epoch.

The Categorical Cross Entropy (CCE) served as the loss function. The Stochastic Gradient Descent (SGD) was performed as the optimizer, with a learning rate of 0.01. And the training strategy of the mini-batch with the size of 32 patches was used on both the DRIVE dataset and the data from Maastricht Study.

## 4. Experimental results

### 4.1. Deep learning network training process

**Figure 5:**
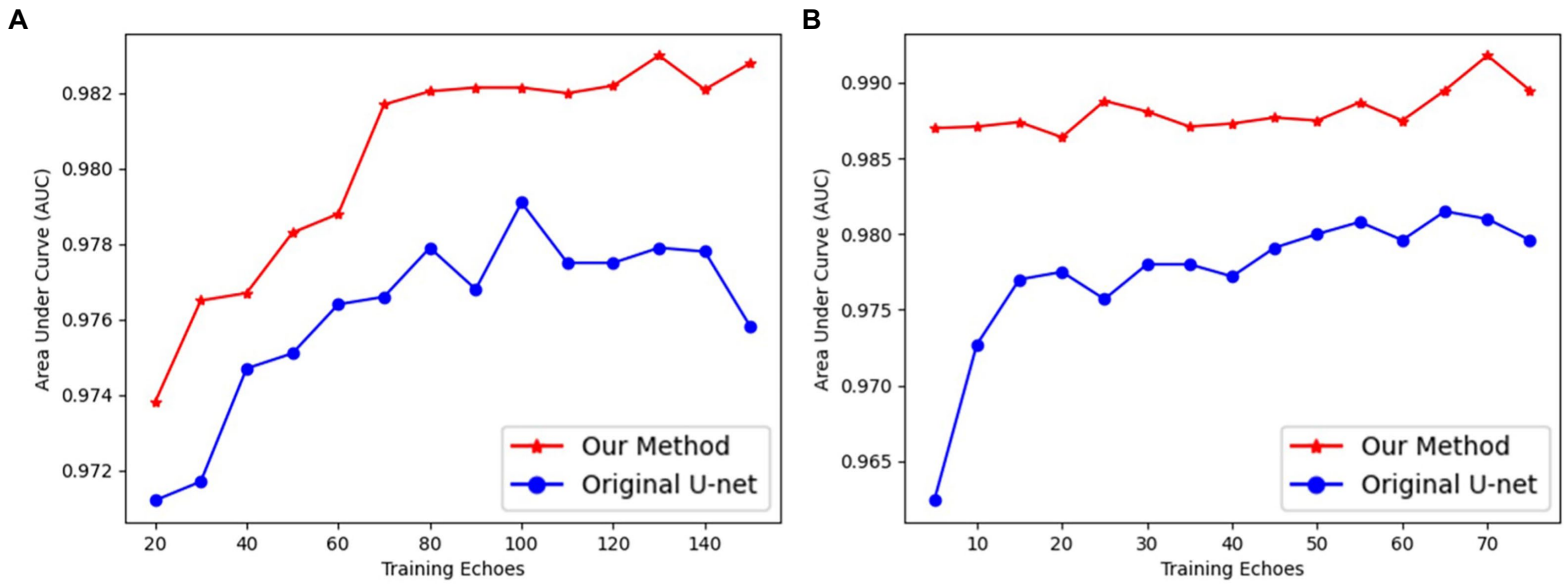
The *AUC* values of proposed UN-LPCOS and original U-Net concerning different training epochs on **(A)** the DRIVE dataset and **(B)** data from the Maastricht study.

Our UN-LPCOS had a faster and more efficient deep-learning training process than the original U-Net. Our method’s network needed fewer epochs to complete training and achieve better performance. In Figure 5, the *AUC* values of each way were calculated with different training epochs. Then the *AUC epoch* − curves were derived, which showed the superior performance of our method on vessel enhancement. When the training was completed, the *AUC* values of our approach were higher than the original U-Net. With the modification of the network architecture, the deep learning model of our method was much easier to train. On the DRIVE dataset, our model needed 70 epochs to complete training, and on the data from Maastricht Study, we needed no more than 10 epochs. For the original U-Net, 100 and 20 epochs were needed on these two datasets, respectively.

### 4.2. Vessel segmentation result

**Figure 6:**
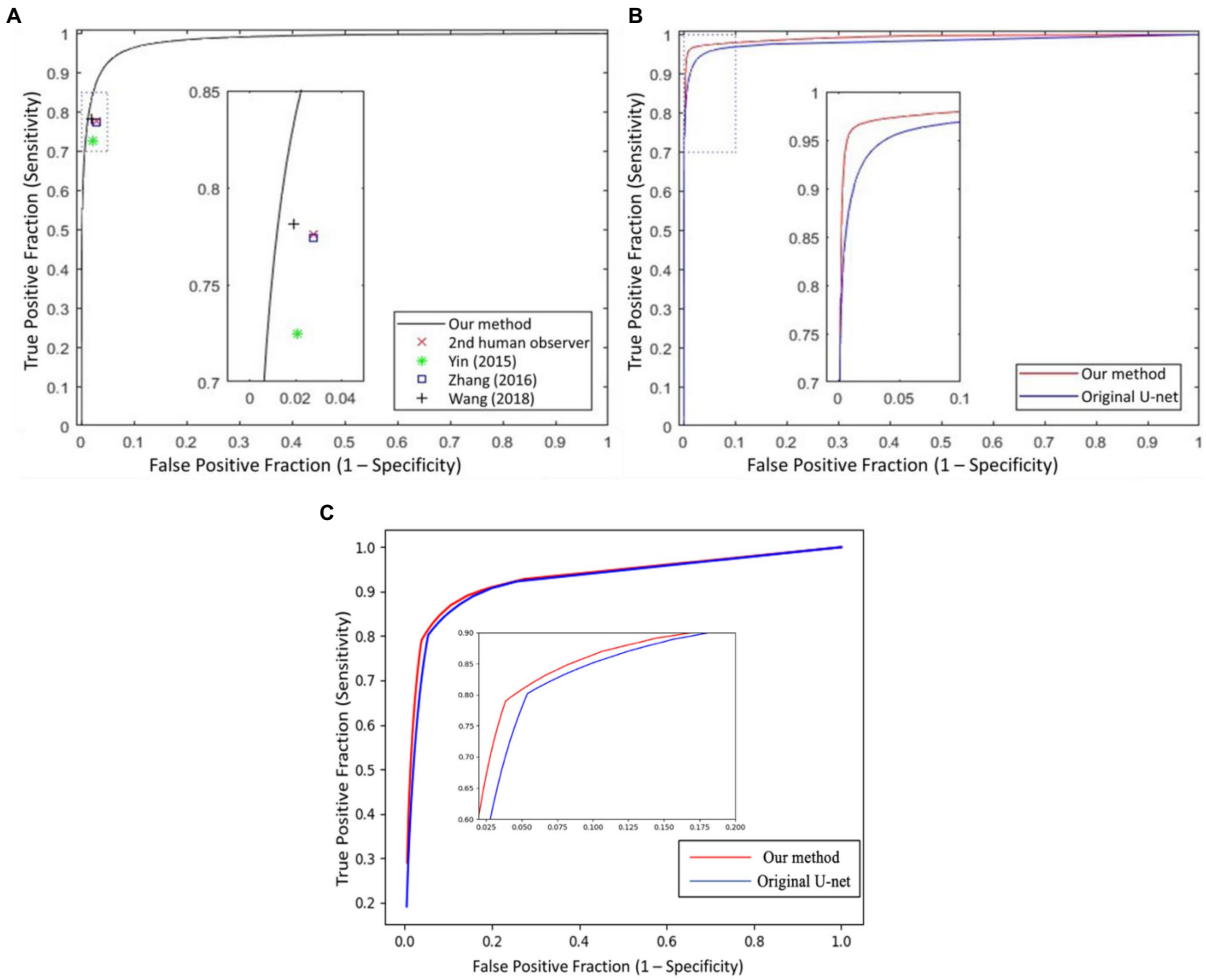
The ROC curves of our UN-LPCOS on **(A)** DRIVE dataset, **(B)** data from the Maastricht study, and **(C)** UoA-DR dataset, compared with the second human observer; the method by Yin et al. (20), Zhang et al. (5), Wang et al. (12), and original U-Net. The dotted box was the critical area which was enlarged and displayed in the figure.

In Figure 6, we presented the ROC curves of our UN-LPCOS on the DRIVE dataset, the data from the Maastricht Study and the UoA-DR dataset. For comparison, the performance of other proposed methods was also depicted. On the Maastricht Study data, our method’s ROC curve was compared with the original U-Net. The evaluation metrics of *Se*, *Sp*, *Acc,* and *AUC* values were presented in Table 1. These metrics, especially *Acc* and *AUC* value, proved the superior performance of our UN-LPCOS. The segmentation performance on small vessels is shown in Table 2. It demonstrated that our method outperformed both datasets’ original U-Net in small vessel segmentation.

**Table 1:** Retinal vessel segmentation results.

	Method	Year	*Se*	*Sp*	*Acc*	*AUC*
**DRIVE dataset**						
	2nd human observer	-	0.7760	0.9724	0.9472	–
Unsupervised method	Al-Diri (8)	2009	0.7282	0.9551	–	–
	Roychowdhury (21)	2015	0.7395	0.9782	0.9494	0.9672
	Azzopardi (3)	2015	0.7655	0.9704	0.9442	0.9614
	Yin (20)	2015	0.7246	0.9790	0.9403	–
	Zhang (5)	2016	0.7743	0.9725	0.9476	0.9636
	Chalakkal (22)		0.7653	0.9735	0.9542	-
Supervised method	Fraz (10)	2012	0.7152	0.9759	0.9430	–
	Orlando (23)	2016	0.7897	0.9684	-	-
	Li (24)	2016	0.7569	0.9816	0.9527	0.9738
	Wang (U-Net) (12)	2018	0.7810	0.9807	0.9536	0.9791
	Xu (25)	2021	–	–	0.9590	0.9713
	Aashis (26)	2020	0.8410	–	0.9633	–
	Our method	2022	**0.8117**	**0.9841**	**0.9658**	**0.9830**
**Data from Maastricht study**						
	U-Net	2018	0.8543	0.9938	0.9842	0.9815
	Our method	2022	**0.9379**	**0.9944**	**0.9905**	**0.9918**
**UoA-DR dataset**						
	U-Net	2018	0.7861	0.9486	0.9366	0.9214
	Our method	2022	**0.8078**	**0.9503**	**0.9396**	**0.9279**

**Table 2:** Small vessel segmentation results.

Method	Year	*Sp*	*Se^sv^*	*AUC^sv^*
**DRIVE dataset**				
U-Net	2018	0.9851	0.6552	0.9502
Our method	2022	**0.9851**	**0.6757**	**0.9665**
**Data from Maastricht study**				
U-Net	2018	0.9943	0.6451	0.9566
Our method	2022	**0.9944**	**0.8475**	**0.9774**
**UoA-DR dataset**				
U-Net	2018	0.9486	0.7734	0.9155
Our method	2022	0.9503	0.7945	0.9216

### 4.3. Results on small vessels

**Figure 7:**
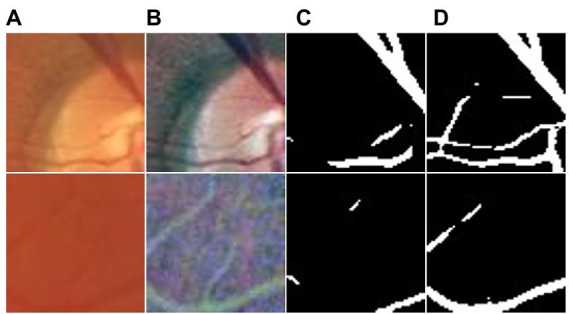
Small vessel segmentation results of our UN-LPCOS and original U-Net. **(A)** Original patches. **(B)** Normalized patches. **(C)** Segmentation results of original U-Net. **(D)** Segmentation results of our UN-LPCOS.

This section highlights the superior performance of the proposed UN-LPCOS in the small vessel segmentation. Figure 7 shows examples of small vessel segmentation produced by the UN-LPCOS and compared with the results derived from the original U-Net. Our method could better segment when faced with minor and low-contrast vessel structures. The example patches were extracted from the DRIVE dataset and the data from Maastricht Study.

**Figure 8:**
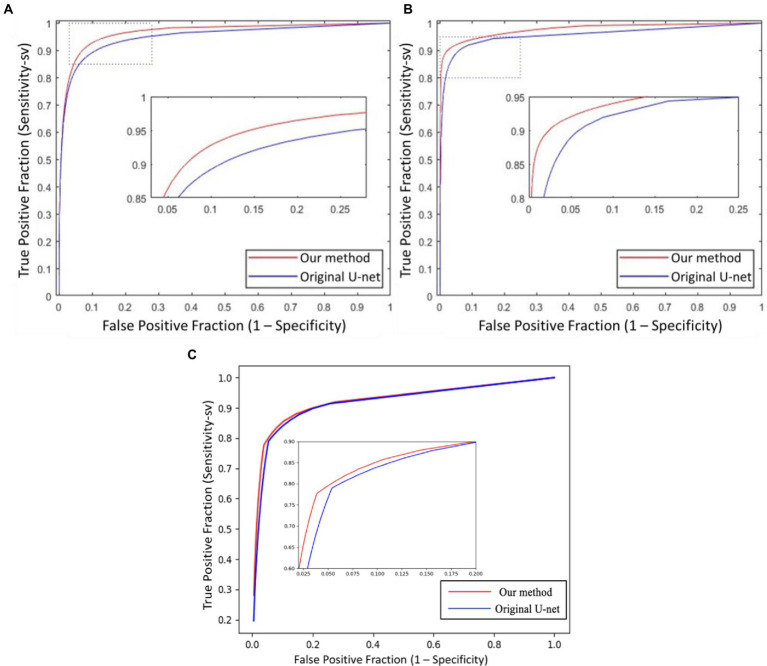
The ROCsv curves of proposed UN-LPCOS and original U-Net derived from the Sesv versus the 1 − Sp with respect to the varying threshold value Th, on **(A)** DRIVE dataset, **(B)** data from the Maastricht study, and **(C)** UoA-DR dataset. The dotted box was the critical area which was enlarged and displayed in the figure.

**Figure 9:**
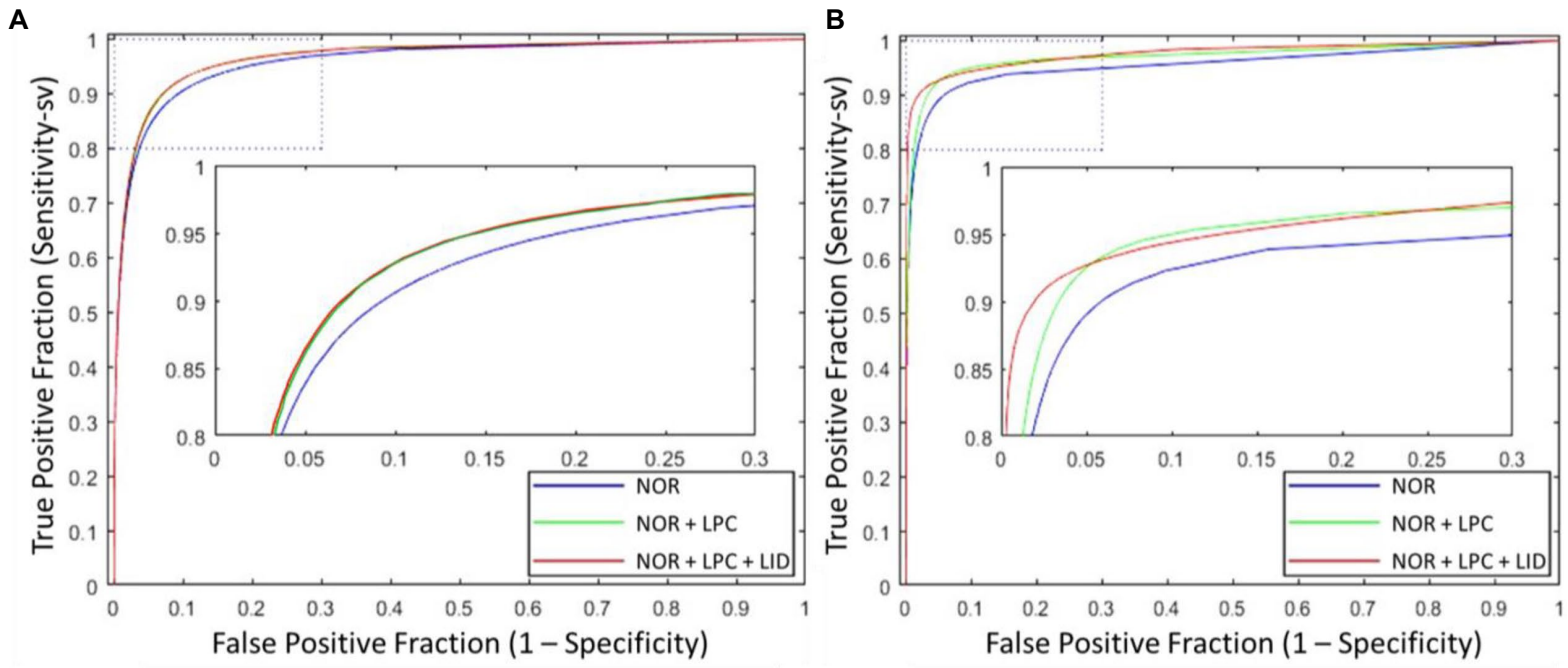
ROC_sv_ curves of proposed UN-LPCOS with different input combinations, which included (1) only normalized retinal images, (2) normalized retinal images and LPC results, (3) normalized retinal images, LPC and LID-OS enhancement results, on **(A)** DRIVE dataset and **(B)** data from Maastricht Study. The dotted box was the critical area which was enlarged and displayed in the figure.

Besides the effect of different input combinations was also discussed. Figure 8 presents the ROCsv curves of the UN-LPCOS and original U-Net. With the changing segmentation threshold, our method consistently achieved higher *Se_sv_* than the original U-Net on the same *Sp* level. And the ROC_sv_ curves of the original U-Net were surrounded by ours on both two datasets. In Figure 9, we analyzed the effect of different unsupervised methods on the segmentation performance in small vessels. Three different input combinations were involved: 1) only normalized retinal images (NOR), 2) normalized retinal images and LPC enhanced retinal images (NOR+LPC), 3) normalized retinal images, LPC and LID-OS enhanced retinal images (NOR+LPC + LID). And we calculated the ROC_sv_ curves for each model.

On the DRIVE dataset, we could see the significant effect of LPC on small vessel segmentation. Besides, adopting LID-OS also impacted the model’s performance, but it was not so obvious compared with LPC. The ROCsv curves of the NOR+LPC (green curves) and the NOR+LPC + LID (red curves) were almost coincident on the DRIVE dataset. On the data from Maastricht Study, however, both LPC and LID-OS obviously influenced model performance. The ROCsv curve of NOR (blue curve) was surrounded by the NOR+LPC one (green curve), which was surrounded by NOR+LPC + LID (red curve).

### 4.4. Result summary

The ROC curve of our UN-LPCOS on the DRIVE dataset is presented in Figure 6A. It showed that our method outperformed other vessel segmentation methods listed in this paper. Compared with the original U-Net, our method also had a superior performance on the data from Maastricht Study and the data from UoA-DR (shown in Figures 6B,C. A more detailed evaluation is presented in Table 1. On the DRIVE dataset, compared with the Se and Sp of some of the best-unsupervised methods (0.7246 and 0.9790 for Yin et al. (20), 0.7743 and 0.9725 for Zhang et al. (5), 0.7653 and 0.9735 for Chalakkal et al. (22)), our method obtained better performance (0.8117 and 0.9841). Besides, we got the highest Acc and AUC value than other supervised methods (0.9658 and 0.9830) on the DRIVE dataset. Compared with the original U-Net, the performance of our modified version achieved significant improvement, with 0.0307 higher in Se and 0.0024 higher in Sp on the DRIVE dataset; 0.0836 higher in Se at the same specificity level on the data of the Maastricht Study;0.0214 higher in Se and 0.0017 higher in Sp on the UoA-DR dataset.

Our method obtained remarkable performance on the small vessel segmentation. We achieved 0.0205, 0.2024 and 0.0211 higher Sesv on the DRIVE dataset, the data from Maastricht Study and UoA-DR dataset than the original U-Net with the same specificity level. Besides, our method produced 0.0163, 0.0208 and 0.0061 higher AUCsv values than the original U-Net on the 3 datasets involved. The comparison of different input combinations was presented in Figure 9, and we could see the effect of other unsupervised methods on the model’s performance of small vessel segmentation. The introduction of LPC significantly improved the *AUC_sv_* value on the DRIVE dataset (0.9598 for the input of NOR and 0.9664 for the NOR+LPC) and the data from the Maastricht Study (0.9557 for the information of NOR and 0.9710 for the NOR+LPC). The LID-OS also had a positive effect, but it was much more limited (0.9664 for the NOR+LPC and 0.9665 for the NOR+LPC + LID on the DRIVE dataset 0.9710 for the NOR+LPC and 0.9774 for the NOR+LPC + LID on the data from Maastricht Study).

## 5. Discussion

### 5.1. Global segmentation performance analysis

The global vessel segmentation performance of different methods is presented in Table 1, and the ROC curves of our proposed UN-LPCOS were showed in Figure 6. The global evaluation metrics and ROC curves demonstrated the outstanding performance of our method. More specifically, compared with other proposed methods, our UN-LPCOS achieved higher *Acc* and *AUC* values on both the DRIVE dataset and the data from Maastricht Study. Besides, the ROC curve of our plan was above all other methods involved in the DRIVE dataset, and on the data from Maastricht Study, our method’s ROC curve surrounded the original U-Net’s.

The UN-LPCOS modified the network structure of the state of art method and adopted the unsupervised methods of LPC and LID-OS for the enhancement of small and complex vessel structures. Therefore, we maintained the excellent results of primary retinal vessel segmentation and improved the ability of small vessel segmentation in the meantime.

However, the small vessels accounted for only a tiny proportion of the retinal image pixels, and the advance of their segmentation did not impact the global result a lot. Thus, the improvement of global performance seemed very limited, especially for the *AUC* value (0.9791 for Wang et al. (12) and0.9830 for proposed UN-LPCOS). Since the retinal image from the Maastricht Study contained more small vessels, the improvement on it was more significant.

### 5.2. Small vessel segmentation performance analysis

The segmentation of small and complex vessel structures is a challenging task. Small retinal vessels are usually a few pixels wide and have low contrast with the background. Therefore, they usually melt in the background noise and are hard to be detected. Besides, the small vessel structure is only a tiny part of the retinal images, which brings more difficulty to training a deep learning network with high sensitivity on small vessels. We applied the LPC method to the retinal images to deal with these problems, and the small vessel structures were significantly highlighted. To present as many details of vessel structures as possible, a low noise threshold and small Gaussian kernels were used, even though they led to more noise in the LPC results.

To avoid the influence of LPC noise on the vessel segmentation, in addition to the LPC value, we also inputted the LPC orientation of each pixel into the deep learning network. The LPC orientations of a vessel segment were the exact or continuous change, while the orientations of LPC noise were the mess. Based on the contextual LPC orientation information, our deep learning network could distinguish between vessels and noise, and the harsh noise in LPC results would not affect the final segmentation. With the support of the LPC, our method was proved to have higher sensitivity in small vessel segmentation.

Moreover, we modified the original network structure of U-Net by replacing the max-pooling layer with the convolutional layer to reduce the loss of image features of small vessels and thus improve the model’s ability to identify small vessels.

### 5.3. Limitation

There are still some limitations of the proposed UN-LPCOS, which need to be solved in future work. Although our method achieved exciting performance on the small vessel segmentation, accurate identification of vessel boundaries remained challenging. Since the boundaries of small vessels were usually blurred in retinal images, it took much work to determine the range of vessel much more accurately. To achieve higher sensitivity on small vessels, our method was more inclined to regard a pixel as the vessel during the vessel enhancement; thus, compared with ground truths, our approach tended to expand the range of small vessels. And the vessel width measured through our method was often more significant than that based on manual segmentation. Besides, connectivity is another weakness of our approach. Since we split one retinal image into many patches, conducted vessel segmentation separately, and stitched together, the vessel connectivity at the suture was reduced. In this paper, only two unsupervised methods were considered to improve the ability of deep learning networks on retinal vessel segmentation. And some other methods shall be introduced, and their effect on the segmentation results will be observed in future work.

## 6. Conclusion

In this paper, we proposed a novel retinal vessel segmentation method named UN-LPCOS. It incorporated a modified U-Net structure and adopted two unsupervised vessel enhancement methods, local phase congruency (LPC) and orientation scores (OS), for attention. The LPC is a frequency domain image analysis method, which is sensitive to the small blood vessels even with low contrast in the retinal images. The OS is a multi-orientation image analysis approach and performs well on complex vessel structures. Adopting these two unsupervised methods boosts our method’s outstanding ability in the segmentation of small and complex vessel structures. An evaluation metric, called sensitivity on small vessels (Sesv), was proposed to describe the method’s performance on the small vessel segmentation. Our plan was validated on the DRIVE dataset and the data from Maastricht Study and achieved superior performance compared to all other methods. Besides, our approach showed outstanding ability in detecting small vessels and outperformed the original U-Net on small retinal vessel segmentation.

## Data availability statement

The original contributions presented in the study are included in the article/supplementary material, further inquiries can be directed to the corresponding author.

## Author contributions

XK: methodology, software, and writing—original draft. XX, LF, EK, HC, and YS: writing—review and editing. FH: software, writing—review, and editing. TT: conceptualization, supervision, project administration, and writing—review and editing. All authors contributed to the article and approved the submitted version.

## Funding

This study was funded by Macao Polytechnic University (grant no. RP/FCA-05/2022).

## Conflict of interest

The authors declare that the research was conducted in the absence of any commercial or financial relationships that could be construed as a potential conflict of interest.

## Publisher’s note

All claims expressed in this article are solely those of the authors and do not necessarily represent those of their affiliated organizations, or those of the publisher, the editors and the reviewers. Any product that may be evaluated in this article, or claim that may be made by its manufacturer, is not guaranteed or endorsed by the publisher.
